# Mitochondrial Ca^2+^ signaling is a hallmark of specific adipose tissue-cancer crosstalk

**DOI:** 10.1038/s41598-024-55650-0

**Published:** 2024-04-11

**Authors:** Agnese De Mario, Elisabetta Trevellin, Ilaria Piazza, Vincenzo Vindigni, Mirto Foletto, Rosario Rizzuto, Roberto Vettor, Cristina Mammucari

**Affiliations:** 1 Department of Biomedical Sciences, University of Padua, via U. Bassi 58/B, 35131 Padua, Italy; 2 Internal Medicine Unit, Department of Medicine, Padua University Hospital, via Giustiniani 2, 35128 Padua, Italy; 3 Clinic of Plastic and Reconstructive Surgery, Department of Neurosciences, University of Padua, Padua, Italy; 4 Bariatric Unit, Department of Surgery, Oncology and Gastroenterology, University of Padua, Padua, Italy

**Keywords:** Breast cancer, Gastrointestinal cancer, Mitochondria, Cancer, Cell invasion, Calcium signalling, Cell biology, Cell migration, Cell signalling, Organelles

## Abstract

Obesity is associated with increased risk and worse prognosis of many tumours including those of the breast and of the esophagus. Adipokines released from the peritumoural adipose tissue promote the metastatic potential of cancer cells, suggesting the existence of a crosstalk between the adipose tissue and the surrounding tumour. Mitochondrial Ca^2+^ signaling contributes to the progression of carcinoma of different origins. However, whether adipocyte-derived factors modulate mitochondrial Ca^2+^ signaling in tumours is unknown. Here, we show that conditioned media derived from adipose tissue cultures (ADCM) enriched in precursor cells impinge on mitochondrial Ca^2+^ homeostasis of target cells. Moreover, in modulating mitochondrial Ca^2+^ responses, a univocal crosstalk exists between visceral adipose tissue-derived preadipocytes and esophageal cancer cells, and between subcutaneous adipose tissue-derived preadipocytes and triple-negative breast cancer cells. An unbiased metabolomic analysis of ADCM identified creatine and creatinine for their ability to modulate mitochondrial Ca^2+^ uptake, migration and proliferation of esophageal and breast tumour cells, respectively.

## Introduction

Obesity is a multifactorial chronic relapsing progressive disease. The primary cause of obesity is likely an alteration of the crosstalk between the mechanisms controlling energy balance in the central nervous system and the peripheral energy stores, mainly the adipose organ^1,2^. Qualitative and quantitative changes of the adipose organ architecture and the consequent functional alterations account for the severity of the disease and the development of severe cardiovascular and metabolic complications including a higher risk of developing different types of cancer^3–5^. The adipose tissue (AT) dysfunction “adiposopathy” is mostly characterized by inflammation and fibrosis^6^. We recently suggested to replace the term 'adiposopathy' with “adiponiche dysfunction”^7^. We identified the adiponiche as the main regulator of AT expansion and remodeling. Thus, the adiponiche and the tumour-niche represent similar morpho-functional units which regulate tissue expansion in AT and cancer, respectively. The obesity-altered adiponiche, as for cancer adiponiche, is characterized by changes in adipose-derived stem cells (ADSC), a multipotent mesenchymal progenitors population that can exert local effects on tumour cells and have been shown to promote various hallmark cancer phenotypes, most notably vascularization^8,9^. The adiponiche is also characterized by the presence of immune and inflammatory cells, and of increased extracellular matrix deposition and tissue fibrosis^7^. These mechanisms have important implications in the development of cancer, contributing to cell survival, growth and proliferation, metabolic reprogramming, angiogenesis, cell invasion and metastasis^10^. During cancer progression, cancer-associated adipocytes undergo considerable morphological and functional alterations acquiring a fibroblast-like phenotype^11,12^. As a result, increased secretion of leptin, adiponectin^13,14^ and pro-inflammatory cytokines including IL-6, TNF-α and IGF1 create a favorable environment inducing tumour cells to acquire a phenotype with major invasiveness and aggressiveness^15–17^. Non-peptide white adipose tissue (WAT)-endocrine factors, including steroid hormones and lipids, also modulate extra-cellular matrix remodeling, cancer cell signaling and metabolism. Among small metabolites, creatine is critical for obesity-driven breast cancer progression^18,19^. ADSC can also influence tumour growth, aggressiveness and metastatic sprouting by secreting pro-angiogenetic growth factors, chemokines and by promoting epithelial mesenchymal transition^20^, suggesting the existence of a paracrine action of adipose tissue in cancer progression^14,21,22^.

Substantial evidence indicates that metabolic modifications support cancer biology and altered mitochondrial functions are fundamental for tumour progression. Mitochondria have a major impact on virtually all processes linked to oncogenesis, encompassing malignant transformation, tumour progression, including the proliferation of transformed cells, their resistance to adverse microenvironmental conditions, their diversification, their interaction with the tumour stroma and their dissemination. Different subsets of malignant cells exhibit differential metabolic profiles, which are important for their survival and function. Besides exerting central bioenergetic functions, mitochondria control tumour metabolism and calcium homeostasis. Mitochondrial Ca^2+^ uptake relies on the activity of a highly selective channel, the mitochondrial calcium uniporter (MCU)^23,24^. Mitochondrial Ca^2+^ uptake occurs in response to physiological stimuli, which trigger the release of Ca^2+^ from intracellular stores, mainly the endoplasmic reticulum (ER). The proximity of the ER to mitochondria in specific sites of close juxtaposition permits the generation of high [Ca^2+^] microdomains at the mouth of Ca^2+^ entry^25^. Ca^2+^ rapidly enters the mitochondrial matrix by virtue of an electrogenic mechanism driven by the large voltage generated across the inner mitochondrial membrane (IMM).

MCU modulation plays multiple roles in cancer progression. On one hand, mitochondrial Ca^2+^ overload is responsible for apoptotic cell death, and reduced MCU activity has been associated with increased cancer cell survival^26,27^. On the other side, MCU activity is required for cancer cell migration, tumour growth, and metastasis formation^28–32^. In the triple negative breast cancer cell line MDA-MB-231, MCU silencing or pharmacological inhibition decreases colony formation and cell migration with a mechanism that requires mitochondrial reactive oxygen species (mROS) and hypoxia-inducible factor 1-alpha (HIF1-α) activity as downstream effectors of mitochondrial Ca^2+^^28^.

We previously reported that the presence of peritumoural adipose tissue in esophageal adenocarcinoma influences the migration and adhesion of cancer cells by exerting a paracrine effect^14,21^. Moreover, obesity is a strong predictor of breast cancer and is associated with more advanced disease, including larger size, higher-grade, lymph node positivity, development of visceral metastases, lower distant disease-free interval and overall survival^33–35^. Cytokines and adipokines are the best characterized adipose tissue-released factors and increasing consensus points to a preferential response of tumours to the specific adjacent adipose tissue. However, most of the molecular targets of this crosstalk are still unknown. Moreover, whether additional molecules participate to the interactions between adipose tissue and tumour is unclear.

Here, we aimed to uncover the mechanisms of the interaction between adipose tissue of specific sites and the cancer cells of organs surrounded by these specific fat depots. We studied the effect of adipose derived conditioned medium (ADCM) isolated from subcutaneous and visceral adipose tissues of donor patients on mitochondrial Ca^2+^ homeostasis in cell models of triple negative breast cancer (MDA-MB-231) and esophageal adenocarcinoma (OE33). Our study indicates a univocal interaction between the progenitor fraction of the resident adipose tissue and the related cancer cell line and identifies creatine and creatinine as adipocyte progenitors-released metabolites impinging on mitochondrial Ca^2+^ uptake and growth of OE33 and in MDA-MB-231 cells, respectively.

## Results

### Conditioned media of adipose tissue enriched in precursor cells increase mitochondrial Ca^2+^ uptake in cancer cells

We wondered whether adipose-tissue released factors impinges on mitochondrial Ca^2+^ uptake in tumour cells. To this aim, we tested adipose-derived conditioned media (ADCM) of primary cell cultures isolated from either subcutaneous or visceral adipose tissue of different donors (SC- and V-ADCM, respectively). Moreover, we discriminated between ADCM of cultures enriched in adipose cell precursors from those of fully differentiated, mature adipocytes (ADCM-P and -D, respectively). To measure Ca^2+^ transients in cancer cells, we used the Ca^2+^-sensitive recombinant photoprotein aequorin targeted to the mitochondrial matrix (mitAEQ) or localized in the cytoplasm (cytAEQ). This approach allowed us to test whether ADCM could affect directly and specifically mitochondrial Ca^2+^ uptake upon ER Ca^2+^ store release with minimal interference of upstream cytosolic Ca^2+^ signals. To induce the release of Ca^2+^ from the ER store, cancer cells expressing mitAEQ or cytAEQ were treated with an InsP_3_-generating agonist. The effects of ADCM-P and ADCM-D on intracellular Ca^2+^ signaling was analyzed in OE33 and MDA-MB-231 cells after 48 h of incubation in serum-free media. No sign of cell death was detected.

**Figure 1:**
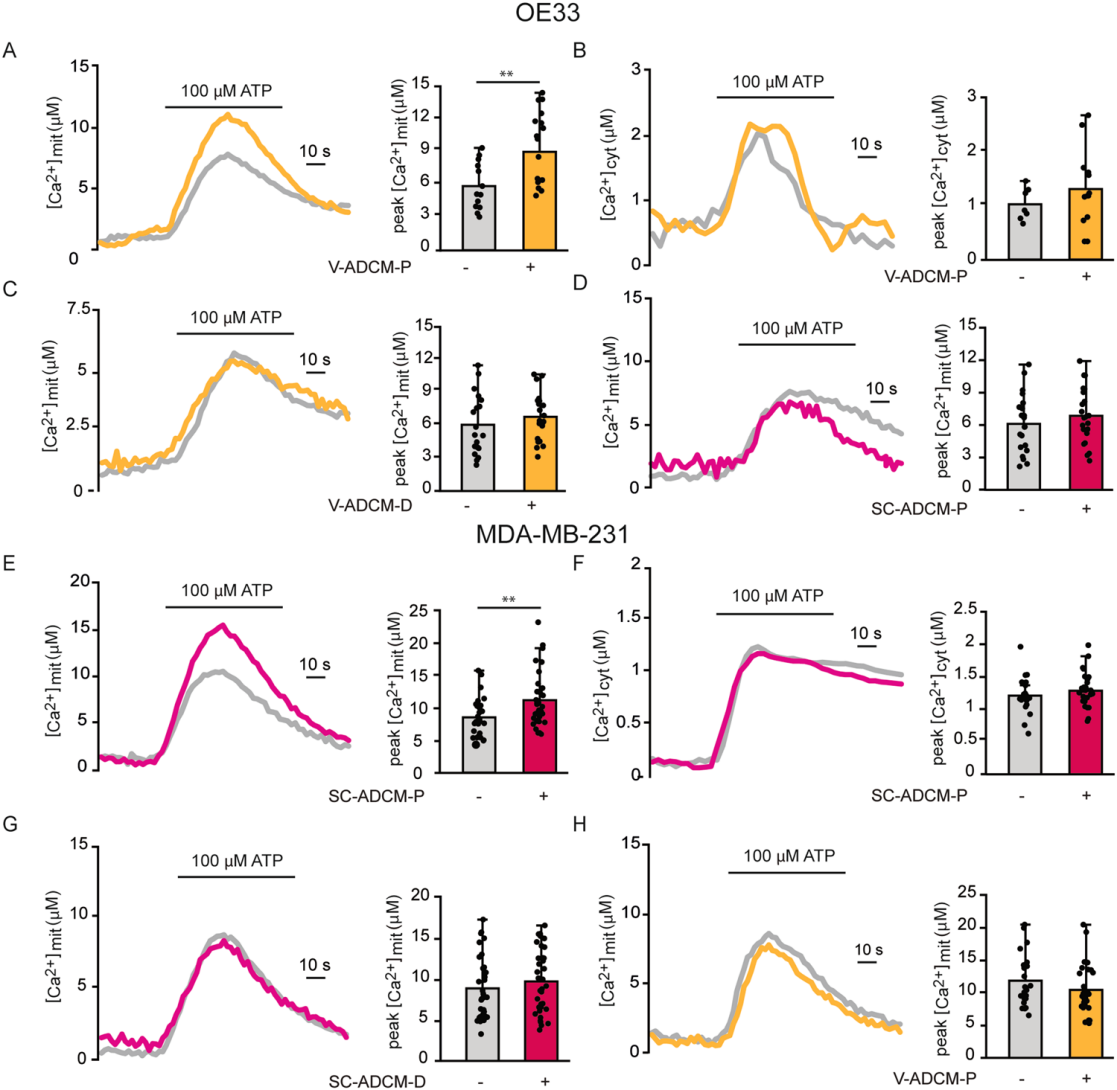
Conditioned media of adipose tissue enriched in precursor cells increase mitochondrial Ca^2+^ uptake in cancer cells. (**A**) Agonist-induced mitochondrial Ca^2+^ uptake in OE33 cells upon V-ADCM-P. Cells were treated with V-ADCM-P for 48 h. Ca^2+^ release from intracellular store was induced by ATP treatment. Left: representative traces. Right: mean [Ca^2+^]mit peaks. (**B**) Agonist-induced cytosolic Ca^2+^ transients in OE33 cells upon V-ADCM-P. Cells were treated with V-ADCM-P for 48 h. Ca^2+^ release from intracellular store was induced by ATP treatment. Left: representative traces. Right: mean [Ca^2+^]cyt peaks. (**C**) Agonist-induced mitochondrial Ca^2+^ uptake in OE33 cells upon V-ADCM-D. Cells were treated with V-ADCM-D for 48 h. Ca^2+^ release from intracellular store was induced by ATP treatment. Left: representative traces. Right: mean [Ca^2+^]mit peaks. (**D**) Agonist-induced mitochondrial Ca^2+^ uptake in OE33 cells upon SC-ADCM-P. Cells were treated with SC-ADCM-P for 48 h. Ca^2+^ release from intracellular store was induced by ATP treatment. Left: representative traces. Right: mean [Ca^2+^]mit peaks. (**E**) Agonist-induced mitochondrial Ca^2+^ uptake in MDA-MB-231 cells upon SC-ADCM-P. Cells were treated with SC-ADCM-P for 48 h. Ca^2+^ release from intracellular store was induced by ATP treatment. Left: representative traces. Right: mean [Ca^2+^]mit peaks. (**F**) Agonist-induced cytosolic Ca^2+^ transients in MDA-MB-231 cells upon SC-ADCM-P. Cells were treated with SC-ADCM-P for 48 h. Ca^2+^ release from intracellular store was induced by ATP treatment. Left: representative traces. Right: mean [Ca^2+^]cyt peaks. (**G**) Agonist-induced mitochondrial Ca^2+^ uptake in MDA-MB-231 cells upon SC-ADCM-D. Cells were treated with SC-ADCM-D for 48 h. Ca^2+^ release from intracellular store was induced by ATP treatment. Left: representative traces. Right: mean [Ca^2+^]mit peaks. (H) Agonist-induced mitochondrial Ca^2+^ uptake in MDA-MB-231 cells upon V-ADCM-P. Cells were treated with V-ADCM-P for 48 h. Ca^2+^ release from intracellular store was induced by ATP treatment. Left: representative traces. Right: mean [Ca^2+^]mit peaks. Data are presented as mean ± SD. ∗∗*p* < 0.01, Student’s t test.

We first evaluated intracellular Ca^2+^ signaling in the esophageal adenocarcinoma cell line OE33 upon treatment with different ADCM. In OE33 cells, 48h incubation with V-ADCM-P increased mitochondrial Ca^2+^ upon ATP-induced ER Ca^2+^ release (Fig. 1A), and this effect was specific for mitochondria, as cytosolic Ca^2+^ transients were unaffected (Fig. 1B). We wondered whether also ADCM from fully differentiated visceral adipocytes exert any effect on Ca^2+^ signaling in cancer cells. However, V-ADCM-D did not increase mitochondrial Ca^2+^ uptake in OE33 cells (Fig. 1C). Thus, OE33 cells were sensitive to one or more factors present in the ADCM obtained by precursor cell cultures, and these factors were absent or inactive in the ADCM of mature adipocytes. We then tested whether SC-ADCM-P exerted a similar effect on mitochondrial Ca^2+^ uptake. However, SC-ADCM-P did not affect mitochondrial Ca^2+^ in OE33 cells (Fig. 1D), indicating a specific interaction between the precursors of visceral adipose tissue and esophageal cancer cells.

We wished to know whether ADCM would alter intracellular Ca^2+^ homeostasis also of triple-negative breast cancer cells. In MDA-MB-231 cells, 48h treatment with SC-ADCM-P increased mitochondrial Ca^2+^ uptake (Fig. 1E) but not cytosolic Ca^2+^ transients (Fig. 1F), indicating a specific effect on mitochondria. On the other hand, SC-ADCM-D did not exert any effect on MDA-MB-231 mitochondrial Ca^2+^ uptake, indicating the presence of specific molecules in the precursors ADCM (Fig. 1G). Finally, treatment with V-ADCM-P did not exert any effect on mitochondrial Ca^2+^ signaling in MDA-MB-231 cells (Fig. 1H), indicating a specific effect of subcutaneous adipose tissue-released factors on breast cancer cells.

Overall these data suggest that adipocyte precursor cells, but not differentiated adipocytes, release specific factors that positively and specifically modulate mitochondrial Ca^2+^ signaling in cancer cells, and that a specific relationship underlies the response of cancer cells to the adjacent adipose tissue.

**Figure 2:**
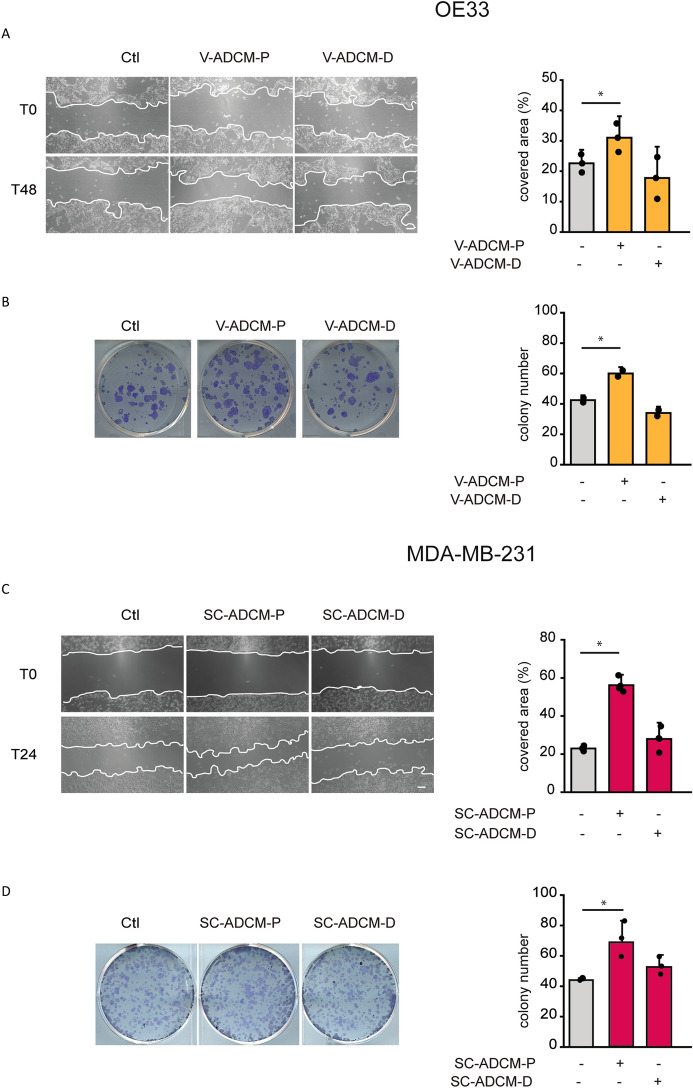
Conditioned media of adipose tissue enriched in precursor cells increase migration and clonogenic potential of cancer cells. (**A**) OE33 cell migration upon treatment with either V-ASCM-P or V-ADCM-D. Cells at 90% confluency were treated with either V-ADCM-P or V-ADCM-D and a linear scratch was made (T0 time point). 48 h after scratching (T48), the area covered by cells was measured and expressed as percentage relative to T0. Left: representative images. Right: quantification. Scale bar, 100 µm. (**B**) OE33 clonogenic potential upon either V-ASCM-P or V-ADCM-D treatments. 10^3^ cells/well were seeded in a 6-well plate. 24 h later, cells were incubated with either V-ASCM-P or V-ADCM-D. 7 days later, colonies containing ≥ 30 cells were counted. Left: representative images. Right: quantification. (**C**) MDA-MB-231 cell migration upon treatment with either SC-ASCM-P or SC-ADCM-D. Cells at 90% confluency were treated either with SC-ADCM-P or SC-ADCM-D and a linear scratch was made (T0 time point). 24 h after scratching (T24), the area covered by cells was measured and expressed as percentage relative to T0. Left: representative images. Right: quantification. Scale bar, 100 µm. (**D**) MDA-MB-231 clonogenic potential upon treatment with either SC-ASCM-P or SC-ADCM-D. 10^3^ cells/well were seeded in a 6-well plate. 24 h later, cells were incubated with either SC-ASCM-P or SC-ADCM-D. 7 days later, colonies containing ≥ 30 cells were counted. Left: representative images. Right: quantification. Data are presented as mean ± SD. ∗*p* < 0.05, one-way ANOVA.

Tumour metastasis is the main cause of esophageal cancer mortality^36^. Thus, we tested whether V-ADCM-P could affect OE33 cell migration and clonogenicity. We measured the capability of OE33 cells to migrate to a wound after 48 h of treatment with V-ADCM-P. In parallel we tested also the effect of V-ADCM-D. Compared to control cells, V-ADCM-P increased OE33 migration while V-ADCM-D did not (Fig. 2A). We next evaluated the effect of V-ADCM-P and V-ADCM-D on OE33 colony formation. After 7 days of treatment, the number of colonies was increased in OE33 cells treated with V-ADCM-P but not with V-ADCM-D (Fig. 2B).

We previously demonstrated that MCU deletion hampers triple-negative breast tumour growth and metastasis formation in vivo, and that MCU silencing or inhibition decreases colony formation and migration of MDA-MB-231 cells^28,55^. Thus, we tested the effect of SC-ADCM-P on these parameters. SC-ADCM-P increased both the cell migration and the clonogenic potential of MDA-MB-231 cells, while SC-ADCM-D did not (Fig. 2C–D).

These data indicate that mitochondrial Ca^2+^ signaling, specifically induced by ADCM-P (either visceral or subcutaneous), impinges on esophageal or breast cancer cell growth and motility, respectively.

### Unbiased metabolomic analysis identifies adipose-derived secreted metabolites

In search of one or more metabolites that may be responsible of the effects of ADCM-P on mitochondrial Ca^2+^ uptake, we performed an unbiased metabolomic analysis comparing the metabolic profile of precursor cell-derived conditioned media with that of differentiated cells (Supporting data values file). Positive modulators of mitochondrial Ca^2+^ uptake should be enriched in the V- and SC-ADCM-P compared to V- and SC-ADCM-D, respectively.

We chose a cut-off value for the q-values of 2 and we selected the following compounds for further analysis among metabolites that were at least two-fold enriched in the ADCM-P compared to the relative ADCM-D: creatine, taurine, guanidinoacetate, creatinine, carnitine, 4-quinolyncarboxylic acid, 2-quinolyncarboxilic acid, 2-aminoadipic acid, N-acetylspermidine and aspartic acid (Table 1 and Supporting data values file).

**Table 1:** Unbiased metabolomic analysis identifies adipose-derived secreted metabolites.

(A) Selected metabolites enriched in V-ADCM-P vs V-ADCM-D									Fold of Change	Statistical Values			
									Matched Pairs t- Test	Matched Pairs t-Test			
									$\frac{{\text{V - ADCM - P}}}{{\text{V - ADCM - D}}}$	$\frac{{\text{V - ADCM - P}}}{{\text{V - ADCM - D}}}$		Chemical ID	CAS
Pathway Sort Order	Super Pathway	Sub Pathway	Biochemical Name	Platform	Comp ID	KEGG	HMDB	PUBCHEM		p value	q-value		
36	Amino Acid	Alanine and Aspartate Metabolism	Aspartic acid	LC/MS pos early	443	C00049	HMDB0000191	5960	2.08	0.06	0.128	234	56-84-8
527		Creatine Metabolism	Guanidinoacetate	LC/MS pos early	43802	C00581	HMDB0000128	763	4.35	0.0001	0.0019	344	352-97-6
528			Creatine	LC/MS pos early	27718	C00300	HMDB0000064	586	6.67	0.0002	0.0033	1221	57-00-1
529			Creatinine	LC/MS pos early	513	C00791	HMDB0000562	588	2.50	0.0227	0.0735	275	60-27-5
123		Lysine Metabolism	2-aminoadipic acid	LC/MS polar	6146	C00956	HMDB0000510	92136	4.17	0.00002	0.0012	381	542-32-5; 1118-90-7
471		Methionine. Cysteine. SAM and Taurine	Taurine	LC/MS polar	2125	C00245	HMDB0000251	1123	2.33	0.0134	0.0542	512	107-35-7
546		Polyamine Metabolism	N-acetylspermidine	LC/MS pos early	57689	C00612	HMDB0001276	496	3.70	0.00001	0.0019	187	14278-49-0
4301	Cofactors and Vitamins	Nicotinate and Nicotinamide Metabolism	2-quinolinecarboxylic acid	LC/MS pos early	1899	C03722	HMDB0000232	1066	4.17	0.0418	0.1011	182	89-00-9
1933	Lipid	Carnitine Metabolism	Carnitine	LC/MS pos early	15500	C00487 C00308	HMDB0000062	288	4.55	0.0086	0.0416	100000007	461-05-2
6264	Xenobiotics	Chemical	4- quinolinecarboxylic acid	LC/MS neg	63767	C06414		10243	14.29	0.0003	0.0042	100021550	486-74-8

(B) Selected metabolites enriched in SC-ADCM-P vs SC-ADCM-D									Fold of Change	Statistical Values			
									Matched Pairs t- Test	Matched Pairs t-Test			
									$\frac{{\text{SC - ADCM - P}}}{{\text{SC - ADCM - D}}}$	$\frac{{\text{SC - ADCM - P}}}{{\text{SC - ADCM - D}}}$		Chemical ID	CAS
Pathway Sort Order	Super Pathway	Sub Pathway	Biochemical Name	Platform	Comp ID	KEGG	HMDB	PUBCHEM		p value	q-value		
36	Amino Acid	Alanine and Aspartate Metabolism	Aspartic acid	LC/MS pos early	443	C00049	HMDB0000191	5960	3.85	0.0053	0.0126	234	56-84-8
527		Creatine Metabolism	Guanidinoacetate	LC/MS pos early	43802	C00581	HMDB0000128	763	4.00	0.0098	0.0176	344	352-97-6
528			Creatine	LC/MS pos early	27718	C00300	HMDB0000064	586	4.76	0.0006	0.003	1221	57-00-1
529			Creatinine	LC/MS pos early	513	C00791	HMDB0000562	588	5.00	0.1028	0.0943	275	60-27-5
123		Lysine Metabolism	2-aminoadipic acid	LC/MS polar	6146	C00956	HMDB0000510	92136	2.86	0.002	0.0071	381	542-32-5; 1118-90-7
471			Taurine	LC/MS polar	2125	C00245	HMDB0000251	1123	2.04	0.0751	0.0727	512	107-35-7
4301	Cofactors and Vitamins	Nicotinate and Nicotinamide Metabolism	2-quinolinecarboxylic acid	LC/MS pos early	1899	C03722	HMDB0000232	1066	4.76	0.1801	0.118	182	89-00-9
1933	Lipid	Carnitine Metabolism	Carnitine	LC/MS pos early	15500	C00487 C00308	HMDB0000062	288	5.56	0.0134	0.0214	100000007	461-05-2
6264	Xenobiotics	Chemical	4-quinolinecarboxylic acid	LC/MS neg	63767	C06414		10243	5.26	0.000004	0.0003	100021550	486-74-8

(A) Metabolites enriched in V-ADCM-P compared to V-ADCM-D. (B) Metabolites enriched in SC-ADCM-P compared to SC-ADCM-D.

### Creatine positively modulates mitochondrial Ca^2+^ uptake, migration and clonogenicity of OE33 cells

**Figure 3:**
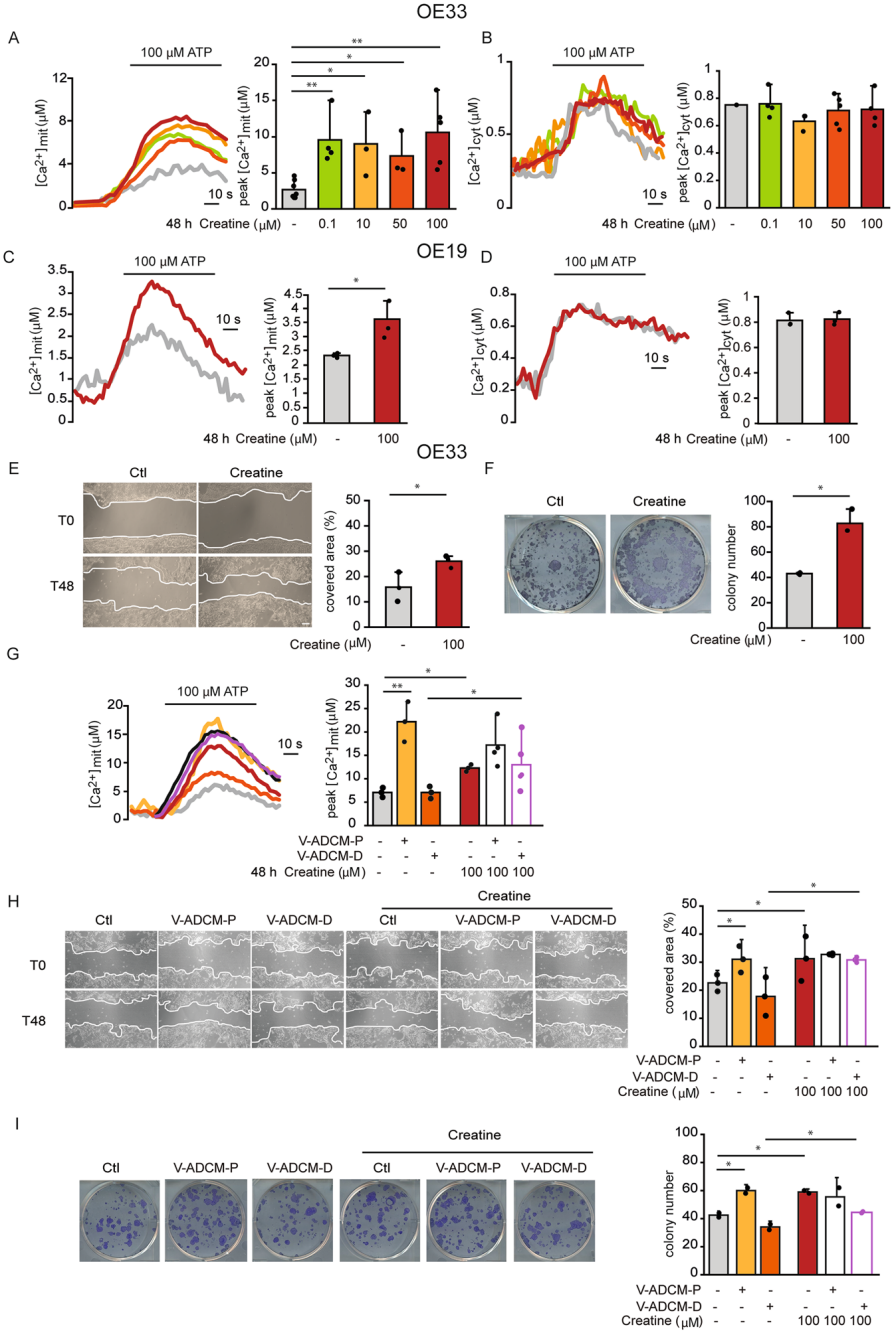
Creatine positively modulates mitochondrial Ca^2+^ uptake, migration and clonogenicity of OE33 cells. (**A**) Agonist-induced mitochondrial Ca^2+^ uptake in OE33 cells upon treatment with creatine. Cells were treated with creatine for 48 h. Ca^2+^ release from intracellular store was induced by ATP treatment. Left: representative traces. Right: mean [Ca^2+^]mit peaks. (**B**) Agonist-induced cytosolic Ca^2+^ transients in OE33 cells upon treatment with creatine. Cells were treated with creatine for 48 h. Ca^2+^ release from intracellular store was induced by ATP treatment. Left: representative traces. Right: mean [Ca^2+^]cyt peaks. (**C**) Agonist-induced mitochondrial Ca^2+^ uptake in OE19 cells upon treatment with creatine. Cells were treated with creatine for 48 h. Ca^2+^ release from intracellular store was induced by ATP treatment. Left: representative traces. Right: mean [Ca^2+^]mit peaks. (**D**) Agonist-induced cytosolic Ca^2+^ transients in OE19 cells upon treatment with creatine. Cells were treated with creatine for 48 h. Ca^2+^ release from intracellular store was induced by ATP treatment. Left: representative traces. Right: mean [Ca^2+^]cyt peaks. (**E**) OE33 cell migration upon creatine treatment. When cells plated in a monolayer reached 90% confluency, they were treated with creatine and a linear scratch was made (T0 time point). 24 h later, creatine-containing medium was replaced. 48 h after scratching (T48), the area covered by cells was measured and expressed as percentage relative to T0. Left: representative images. Right: quantification. Scale bar, 100 µm. (**F**) OE33 clonogenic potential upon creatine treatment. 10^3^ cells/well were seeded in a 6-well plate. 24 h later, cells were incubated with creatine. 7 days later, colonies containing ≥ 30 cells were counted. Left: representative images. Right: quantification. (**G**) Agonist-induced mitochondrial Ca^2+^ uptake in OE33 cells upon treatment with V-ADCM in the presence or absence of creatine. Cells were treated with either V-ADCM-P or V-ADCM-D, with or without creatine for 48 h. Ca^2+^ release from intracellular store was induced by ATP treatment. Left: representative traces. Right: mean [Ca^2+^]mit peaks. (**H**) OE33 cell migration upon treatment with V-ADCM, in the presence or absence of creatine. Cells at 90% confluency were treated with either V-ADCM-P or V-ADCM-D, with and without creatine and a linear scratch was made (T0 time point). 24 h later, creatine-containing media were replaced. 48 h after scratching (T48), the area covered by cells was measured and expressed as percentage relative to T0. Left: representative images. Right: quantification. Scale bar, 100 µm. (**I**) OE33 clonogenic potential upon treatment with V-ADCM, in the presence or absence of creatine. 10^3^ cells/well were seeded in a 6-well plate. 24 h later, cells were incubated with either V-ADCM-P or V-ADCM-D with and without creatine. 7 days later, colonies containing ≥ 30 cells were counted. Left: representative images. Right: quantification. Data are presented as mean ± SD. ∗*p* < 0.05, ∗∗*p* < 0.01, Student’s t test except one-way ANOVA for panels A and B and two-way ANOVA for panels (**G**–**I**).

The selected metabolites were tested for their ability to modulate mitochondrial Ca^2+^ uptake in the OE33 cell line, when added at different concentrations (0.1, 10, 50, 100 µM) for 48 h, i.e. the incubation time in which V-ADCM-P exerted a positive effect. In OE33 cells, creatinine, taurine, guanidinoacetate, carnitine, 4-quinolyncarboxylic acid, 2-aminoadipic acid, N-acetylspermidine and aspartic acid did not exert any effect on mitochondrial Ca^2+^ signaling (Fig. S1A–E, G–I), and 2-quinolyncarboxilic acid reduced mitochondrial Ca^2+^ (Fig. S2F). Thus, none of these metabolites could be responsible for the increase in mitochondrial Ca^2+^ uptake exerted by V-ADCM-P. On the other hand, creatine increased ATP-induced mitochondrial Ca^2+^ uptake at all tested concentrations (Fig. 3A) without affecting cytosolic Ca^2+^ (Fig. 3B). The effect of creatine on Ca^2+^ signaling was confirmed in the esophageal cancer OE19 cell line in which, similarly to OE33 cells, creatine increased mitochondrial Ca^2+^ (Fig. 3C) without affecting cytosolic Ca^2+^ (Fig. 3D). Overall, these data suggest that creatine positively modulates mitochondrial Ca^2+^ signaling in esophageal cancer cell lines.

After verifying that creatine has no effect on OE33 cell proliferation or viability (data not shown), we measured its effect on OE33 migration. Compared to control cells, 48 h treatment with creatine increased OE33 motility (Fig. 3E). We next evaluated the effect of creatine on colony formation. After 7 days of treatment, the number of colonies was increased in creatine-treated OE33 cells (Fig. 3F), mimicking the effect of V-ADCM-P. To evaluate whether the effects induced by creatine on mitochondrial Ca^2+^ uptake, cell migration and clonogenic potential were additive to the effects of V-ADCM-P, we incubated OE33 cells with V-ADCM-P in the presence or absence of creatine (100 µM). Creatine did not exert an additive effect compared to V-ADCM-P alone (Fig. 3G–I).

Next, we wished to know whether creatine repletion in V-ADCM-D was sufficient to rescue the measured parameters. Accordingly, we incubated OE33 cells with V-ADCM-D with or without creatine (100 µM) and we measured mitochondrial Ca^2+^ uptake, migration and colony number. Compared to V-ADCM-D, creatine increased both mitochondrial Ca^2+^ uptake and OE33 cell migration (Fig. 3G, H). On the same line, after 7 days of treatment, creatine rescued the effect of V-ADCM-D on the clonogenicity of OE33 cells (Fig. 3I).

### Creatinine positively modulates mitochondrial Ca^2+^, migration and clonogenicity of MDA-MB-231 cells

We then searched which metabolites enriched in the SC-ADCM-P compared to the SC-ADCM-D (Fig. 2) exert a positive effect on mitochondrial Ca^2+^ signaling in MDA-MB-231 triple-negative breast cancer cells. Differently from OE33 cells, creatine did not affect mitochondria Ca^2+^ in MDA-MB-231 cells (Fig. S2A), indicating a cell-type specificity. Other metabolites, including taurine, guanidinoacetate, carnitine, 4-quinolyncarboxylic acid, 2-aminoadipic acid, N-acetylspermidine, aspartic acid and 2-quinolyncarboxilic acid did not alter intracellular Ca^2+^ homeostasis (Fig. S2B–I).

**Figure 4:**
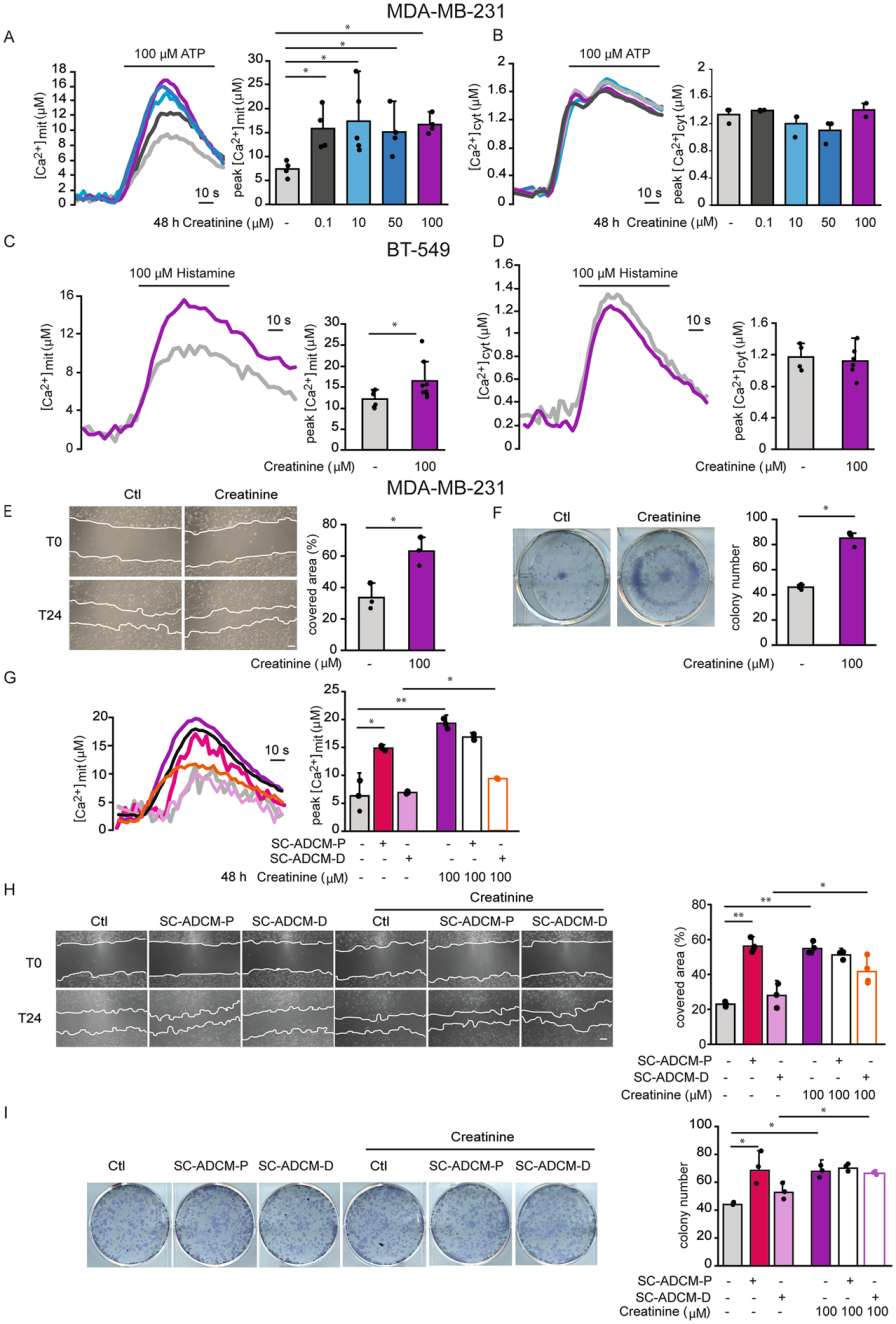
Creatinine positively modulates mitochondrial Ca^2+^ uptake in breast cancer cells. (**A**) Agonist-induced mitochondrial Ca^2+^ uptake in MDA-MB-231 cells upon treatment with creatinine. Cells were treated with creatinine for 48 h. Ca^2+^ release from intracellular store was induced by ATP treatment. Left: representative traces. Right: mean [Ca^2+^]mit peaks. (**B**) Agonist-induced cytosolic Ca^2+^ transients in MDA-MB-231 cells upon treatment with creatinine. Cells were treated with creatinine for 48 h. Ca^2+^ release from intracellular store was induced by ATP treatment. Left: representative traces. Right: mean [Ca^2+^]cyt peaks. (**C**) Agonist-induced mitochondrial Ca^2+^ uptake in BT-549 cells upon treatment with creatinine. Cells were treated with creatinine for 48 h. Ca^2+^ release from intracellular store was induced by ATP treatment. Left: representative traces. Right: mean [Ca^2+^]mit peaks. (**D**) Agonist-induced cytosolic Ca^2+^ transients in BT-549 cells upon treatment with creatinine. Cells were treated with creatinine for 48 h. Ca^2+^ release from intracellular store was induced by ATP treatment. Left: representative traces. Right: mean [Ca^2+^]cyt peaks. (**E**) MDA-MB-231 cell migration upon creatinine treatment. When cells plated in a monolayer reached 90% confluency, they were treated with creatinine and a linear scratch was made (T0 time point). 24 h after scratching (T24), the area covered by cells was measured and expressed as percentage relative to T0. Left: representative images. Right: quantification. Scale bar, 100 µm. (**F**) MDA-MB-231 clonogenic potential upon creatinine treatment. 10^3^ cells/well were seeded in a 6-well plate. 24 h later, cells were incubated with creatinine. 7 days later, colonies containing ≥ 30 cells were counted. Left: representative images. Right: quantification. (**G**) Agonist-induced mitochondrial Ca^2+^ uptake in MDA-MB-231 cells upon treatment with SC-ADCM, in the presence or absence of creatinine. Cells were treated with SC-ADCM-P, SC-ADCM-D with or without creatinine for 48 h. Ca^2+^ release from intracellular store was induced by ATP treatment. Left: representative traces. Right: mean [Ca^2+^]mit peaks. (**H**) MDA-MB-231 cell migration upon treatment with SC-ADCM, in the presence or absence of creatinine. Cells at 90% confluency were treated with SC-ADCM-P, SC-ADCM-D, with or without creatinine and a linear scratch was made (T0 time point). 24 h after scratching (T24), the area covered by cells was measured and expressed as percentage relative to T0. Left: representative images. Right: quantification. Scale bar, 100 µm. (**I**) MDA-MB-231 clonogenic potential upon treatment with SC-ADCM, in the presence or absence of creatinine. 10^3^ cells/well were seeded in a 6-well plate. 24 h later, cells were incubated with either SC-ADCM-P or SC-ADCM-D, with or without creatinine. 7 days later, colonies containing ≥ 30 cells were counted. Left: representative images. Right: quantification. Data are presented as mean ± SD. ∗*p* < 0.05, Student’s t test except one-way ANOVA for panels A and B and two-way ANOVA for panels (**G**–**I**).

Among the selected metabolites, creatinine was the only one to increase ATP-induced mitochondrial Ca^2+^ uptake (Fig. 4A) without affecting cytosolic Ca^2+^ (Fig. 4B) upon 48h treatment. The effect of creatinine on mitochondrial Ca^2+^ was also tested in another model of breast cancer, the BT549 cell line in which, similarly to MDA-MB-231 cells, creatinine increased mitochondrial Ca^2+^ uptake (Fig. 4C) without affecting cytosolic Ca^2+^ transients (Fig. 4D). These results demonstrate that creatinine positively modulates mitochondrial Ca^2+^ signaling in different breast cancer cell lines.

After assessing that 24 h incubation with creatinine does not impinge on MDA-MB-231 cell proliferation and viability (data not shown), we proceeded to investigate its effect on cell migration and clonogenic potential, revealing an increase in both parameters (Fig. 4E–F).

To evaluate whether the effects induced by creatinine on MDA-MB-231 mitochondrial Ca^2+^ uptake, migration and clonogenicity were additive to the effects of SC-ADCM-P, we incubated the cells with SC-ADCM-P in the presence or absence of creatinine (100 µM). The addition of creatinine to SC-ADCM-P did not exert an additive effect on any of these parameters (Fig. 4G–I).

Finally, we checked whether creatinine addition to SC-ADCM-D exerted any effect on mitochondrial Ca^2+^ uptake, migration and colony number. After 48 h of incubation, compared to SC-ADCM-D alone, creatinine addition increased all parameters (Fig. 4G–I).

### Creatine and creatinine increase directly and specifically MCU activity

**Figure 5:**
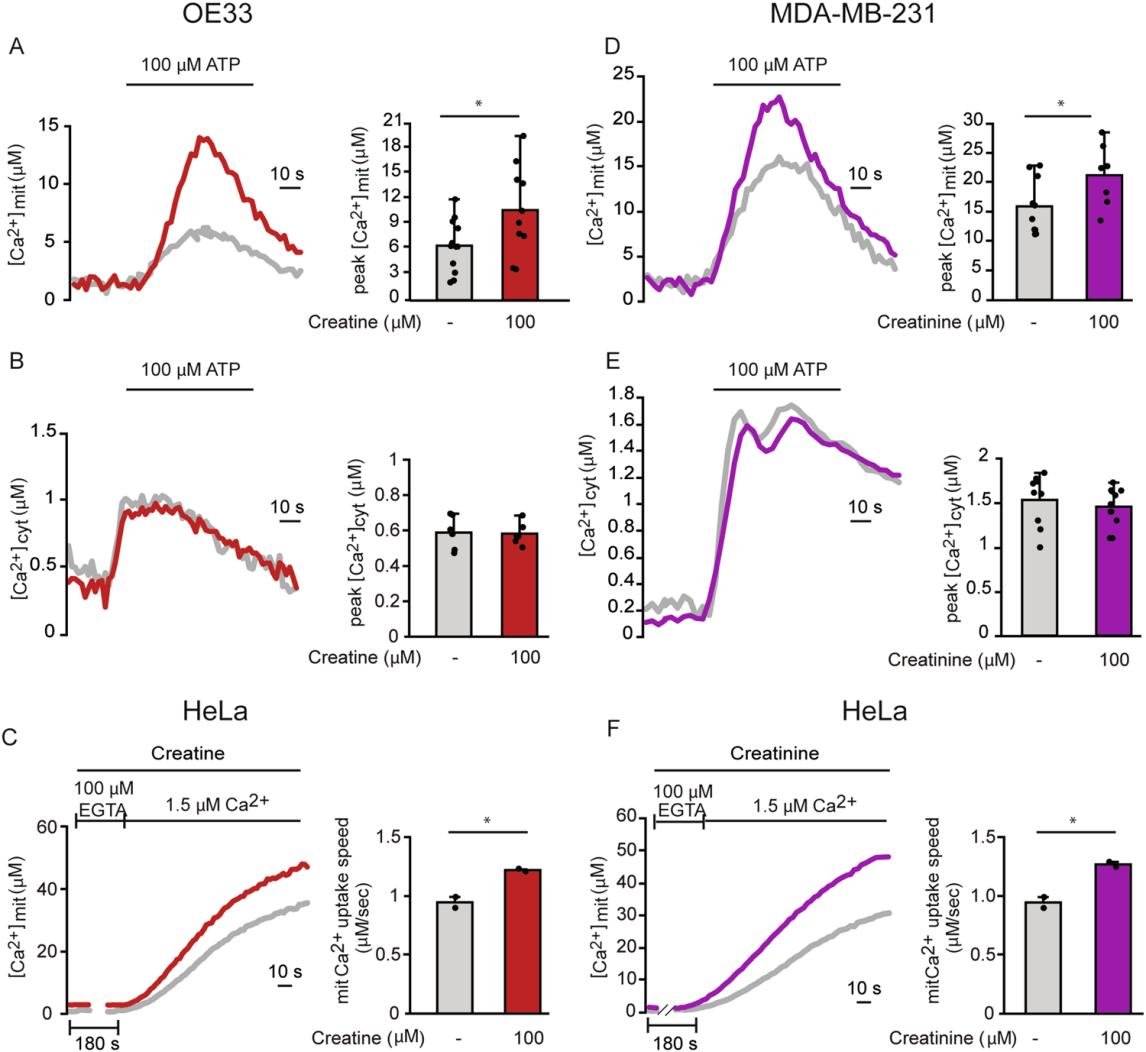
Creatine and creatinine increase directly and specifically MCU activity. (**A**) Agonist-induced mitochondrial Ca^2+^ uptake in OE33 cells. Cells were treated with creatine before (30 s) and during ATP stimulation. Left: representative traces. Right: mean [Ca^2+^]mit peaks. (**B**) Agonist-induced cytosolic Ca^2+^ transients in OE33 cells. Cells were treated with creatine before (30 s) and during ATP stimulation. Left: representative traces. Right: mean [Ca^2+^]cyt peaks. (**C**) Mitochondrial Ca^2+^ uptake measurements in permeabilized HeLa cells. Cells were treated with creatine before (180 s) and during Ca^2+^ perfusion. Left: representative traces. Right: mean [Ca^2+^]mit speed. (**D**) Agonist-induced mitochondrial Ca^2+^ uptake in MDA-MB-231 cells. Cells were treated with creatinine before (30 s) and during ATP stimulation. Left: representative traces. Right: mean [Ca^2+^]mit peaks. (**E**) Agonist-induced cytosolic Ca^2+^ uptake in MDA-MB-231 cells. Cells were treated with creatinine before (30 s) and during ATP stimulation. Left: representative traces. Right: mean [Ca^2+^]cyt peaks. (**F**) Mitochondrial Ca^2+^ uptake measurements in permeabilized HeLa cells. Cells were treated with creatinine before (180 s) and during Ca^2+^ perfusion. Left: representative traces. Right: mean [Ca^2+^]mit speed. Data are presented as mean ± SD. ∗*p* < 0.05, Student’s t test.

Mitochondrial Ca^2+^ uptake increases require activation of the Mitochondrial Calcium Uniporter (MCU), the highly selective channel of the inner mitochondrial membrane responsible for mitochondrial Ca^2+^ entry. We wished to discriminate whether prolonged creatine and creatinine treatment, most likely impinging on transcriptional and /or translational regulation of the MCU complex components, is required, or rather short-term incubation, acting on the MCU complex conformation, is sufficient to increase mitochondrial Ca^2+^ uptake. To this aim, we acutely treated OE33 or MDA-MB-231 cells with creatine and creatinine respectively, and then we stimulated Ca^2+^ release from the ER stores with ATP. In these conditions, creatine increased mitochondrial Ca^2+^ in OE33 cells (Fig. 5A), and creatinine exerted a similar effect in the MDA-MB-231 cell line (Fig. 5D) without affecting cytosolic Ca^2+^ (Fig. 5B, E), indicating that the two metabolites directly modulate the activity of the MCU in their respective target cells.

To further corroborate the specificity and selectivity of creatine and creatinine on the MCU complex, we measured mitochondrial Ca^2+^ uptake in permeabilized HeLa cells in which intracellular Ca^2+^ stores are depleted and mitochondria are exposed to a buffer solution containing a well-defined [Ca^2+^]. Under these conditions, the two metabolites were able to increase mitochondrial Ca^2+^ uptake speed (Fig. 5C, F), indicating a direct activation of the MCU channel.

## Discussion

In breast, esophageal, colon, liver and prostate cancers, obesity represents a poor predictor of clinical outcomes^37,38^ and for breast cancer and esophageal adenocarcinoma an increased adiposity/body fat accumulation is considered a more discriminating risk factor for cancer incidence than the commonly used BMI^39–41^. In this study we show that ADCM derived from cultures containing adipocyte precursor cells (ADCM-P) positively modulate mitochondrial Ca^2+^ signaling and the capability of the recipient tumour cells to migrate and to form colonies. Indeed, when incubated with breast and esophageal cancer cells, ADCM-P increased mitochondrial Ca^2+^ uptake without affecting the overall cytosolic [Ca^2+^]. Interestingly, the tumour cells of different origin were differently susceptible to ADCM-P derived from the visceral adipose tissue of patients undergoing bariatric surgery or from the subcutaneous adipose tissue of patients undergoing plastic surgery. In detail, in breast cancer cells the effects on mitochondrial Ca^2+^ homeostasis are evident when treated with SC-ADCM, while esophageal cancer cells are susceptible only to V-ADCM. These results are particularly interesting since visceral adipose tissue resides in contact with the esophagus, just as the subcutaneous adipose tissue does with the mammary gland (but not vice versa). Regarding the differentiation status of the adipocytes, we observed that the media collected from mature adipocytes affect neither mitochondrial Ca^2+^ uptake, nor migration or clonogenic potential of OE33 and MDA-MB-231 cells. Therefore, specific factors contained in ADCM-P impinge on mitochondrial Ca^2+^ uptake, in a tissue-specific manner.

Previous studies had already shown how conditioned medium of adipose-derived mesenchymal stem cells can have several effects on different type of cells and tissues^39,42^. However, those studies were conducted with conditioned media derived from the heterogenous, multipotent stromal vascular cell population and not from the adipose-specific preadipocytes used in this study.

We further searched which metabolite/s could be responsible for the observed effects. By unbiased metabolic analysis, metabolites involved glycolysis, glutaminolysis, tricarboxylic acid cycle, fatty acid, phospholipid and one-carbon metabolism were increased in visceral and in subcutaneous ADCM-P. Nonetheless, among selected metabolites, only creatine and creatinine increased mitochondrial Ca^2+^ uptake in OE33 and in MDA-MB-231 cells, respectively. Interestingly, creatine and creatinine mimicked the specific effect of visceral and subcutaneous ADCM-P on cancer cells, i.e. creatine increased mitochondrial Ca^2+^ uptake in OE33 and creatinine in MDA-MB-231. Creatine and creatinine respectively modulated mitochondrial Ca^2+^ uptake in additional esophageal and triple negative breast cancer cell lines (OE19 and BT549), suggesting the existence of a general mechanism.

Creatine is a nitrogen-containing organic acid obtained daily from creatine-enriched diet such as fish, poultry and red meat^43^. In addition, creatine is synthesized in the kidneys and liver from glycine, arginine, and methionine through a two-step process involving the methylation of guanidinoacetate via S-adenosylmethionine (SAM), forming creatine and S-adenosylhomocysteine (SAH). Creatine is then transported by the creatine transporter SLC6A8 from the circulation into target cells, where it is phosphorylated by creatine kinase to form phosphocreatine. Finally, creatine slowly and spontaneously cyclizes to creatinine, which is eliminated in the urine^43^.

Our data on the effects of creatine and creatinine in cancer cell growth and migration are in line with previous findings indicating a role for creatine in facilitating obesity-accelerated cancer^19,47^ and provide new information about the possible involvement of creatinine in cancer progression. To the best of our knowledge only an indirect link between creatinine and cancer has been reported so far. In fact, the only available data consist in retrospective studies in which high plasma levels of creatinine are associated with a worse prognosis in vulvar cancer^44^, colorectal cancer^45^ and ovarian cancer^46^. In hepatocellular carcinoma (HCC) patients, creatine level is significantly increased in urine, and correlates with the clinical stage of HCC^47^. Moreover, women with higher levels of creatine and of its metabolite creatinine in plasma have a higher risk of breast cancer^48^. In addition, a retrospective cohort study positively correlated serum creatine and creatinine levels with poor disease-specific and overall survival in 170 patients with invasive vulvar cancer^44,46^. However, on the contrary, in a sarcoma mouse model, creatine content gradually decreased with the progression of malignancy^49^.

As for the mechanism, creatine and creatinine did not alter cytosolic Ca^2+^ homeostasis and positively modulated mitochondrial Ca^2+^ uptake rate in permeabilized HeLa cells, suggesting a direct and specific modulation of the MCU. While V-ADCM-P is more enriched in creatine than creatinine, in SC-ADCM-P creatine and creatinine are enriched in similar amounts. Therefore, the specificity of creatine and creatinine could not be explained by their different enrichment in the different media, but rather by cell-type differences. Finally, a decreased/lower trend in creatine and creatinine in ADCM-D compared to ADCM-P could explain the absence of effects of ADCM-D on mitochondrial Ca^2+^ in both cancer cell lines, as corroborated by the fact that metabolite addition to ADCM-D mimicked the effect of ADCM-P. In conclusion, our findings indicate that visceral and subcutaneous adipose tissues could modulate mitochondrial Ca^2+^ homeostasis in esophageal and breast cancer cells in a depot-specific and cell type-exclusive manner involving creatine and creatinine, respectively, which in turn sustain cancer progression.

## Methods

### Patients

Adipose tissue was isolated from 19 patients aged 18–60 years with a BMI > 40.0 kg/m^2^ or with BMI between 35.0 and 39.9 kg/m^2^ and co-morbidities, in whom surgically induced weight loss was expected to improve the disorder^50^, enrolled in the Center for the Study and the Integrated Treatment of Obesity of the University Hospital of Padova. In particular, visceral adipose tissue was isolated from obese patients who underwent bariatric surgery (laparoscopic sleeve gastrectomy) for weight loss (n = 9), whereas subcutaneous adipose tissue was isolated from formerly obese patients who underwent plastic surgery (abdominoplasty) after bariatric surgery-induced weight loss (n = 10).

### ADSC culture, differentiation and conditioned media collection

ADSC were isolated from visceral and subcutaneous adipose tissue of 19 patients undergoing bariatric or plastic surgery, respectively. Briefly, freshly isolated adipose tissue was collected from omental or abdominal depot and separated from major vessels and fibers, minced and digested with 1 mg/mL collagenase type II (Sigma-Aldrich, St Louis, MO, USA) in DMEM/F12 at 37 °C for 1 h. Cell suspension containing stromal vascular fraction (SVF) was centrifuged (350 g, 8 min), pelleted and resuspended in erythrocyte-lysing buffer, washed in DMEM/F12, filtered with a 100 μm cell strainer, centrifuged (350 g, 8 min), washed again in DMEM/F12 and seeded in DMEM/F12 supplemented with 10% FBS (5*10^5^ cells/well in 24-well plates). After 24 h cells were washed twice in warm sterile PBS and the medium was replaced with low-glucose DMEM free from both phenol red and FBS. After 24 h, ADCM was collected (ADCM-P) and the medium was replaced with adipogenic medium: DMEM/F12 supplemented with 33 μM biotin, 17 μM pantothenate, 10 μg/mL human transferrin (Sigma-Aldrich), 66 μM insulin (Lilly Research, Indianapolis, IN, USA), 100 μM dexamethasone, 1 μM T3, 0.25 μM 3-isobutyl-1-methylxanthine (IBMX; Sigma-Aldrich) and 10μM rosiglitazone. After 3 days rosiglitazone and IBMX were removed and the medium was changed three times per week until complete differentiation was obtained (after 14 days), at which the medium was replaced with low-glucose DMEM free from both phenol red and FBS. After 24 h ADCM derived from fully differentiated mature adipocytes (ADCM-D) was collected. Preadipocytes maintained in DMEM/F12 alone were used as controls (undifferentiated cells). ADCM-P and ADCM-D were instantly frozen in liquid nitrogen in an attempt to maintain reproducible handling and storage procedures for each patient.

### Cell lines

HeLa cells were purchased from ATCC and cultured in Dulbecco’s modified Eagle’s medium (DMEM) (Gibco). OE33 and OE19 were purchased from Sigma and ATCC respectively and cultured in RPMI 1640 medium (Gibco). MDA-MB-231 and BT-549 cells were purchased from ATCC and cultured in DMEM-F12 (Gibco) and in DMEM (Gibco) respectively. All media were supplemented with 10% foetal bovine serum, 150U/mL streptomycin, 200U/mL penicillin and 2mM glutamine (Gibco). Cells were maintained at 37 °C and 5% CO_2_ in incubator.

### Plasmids and transfection

Plasmids encoding mitochondria targeted aequorin (mitAEQ)^51^ and cytosolic aequorin (cytAEQ)^52^ were previously described. HeLa cells were transfected with a standard Ca^2+^ phosphate procedure as already described^24,53^. OE33, OE19, MDA-MB-231 and BT549 were transfected with LipofectamineTM 2000 transfection reagent (Invitrogen).

### Metabolites

Creatine (Sigma Aldrich, C0780), creatinine (Sigma Aldrich, 1052060050), taurine (Sigma Aldrich, T8691), guanidine acetate (Sigma Aldrich, 50,920), carnitine (Sigma Aldrich, C9500), 4-quinolinecarboxylic acid (Sigma Aldrich, 174823), quinoline-2,4-dicarboxilic acid (Sigma Aldrich, 8151030025), DL-2-aminoadipic acid (Sigma Aldrich, A0637), N1-acetylspermidine (Cayman Chemical 9001535), N-acetyl aspartic acid (Sigma Aldrich, 00920) were used.

### Calcium measurements

For measurements of cytosolic Ca^2+^ and mitochondrial Ca^2+^, OE33, OE19, MDA-MB-231, BT549 and HeLa cells were grown on 13-mm round glass coverslips and transfected as previously described^54,55^. For some experiments, the day of the transfection cells were treated with ADCM-P or ADCM-D diluted 1:1 in serum-free medium. When needed, cells were treated with creatine or creatinine in serum-free medium. 48 h later, cells were incubated with 5 mM coelenterazine for 1 h in KRB at 37 °C supplemented with 1 mM CaCl_2_. In experiments with intact cells, those were transferred to the perfusion chamber where Ca^2+^ transients were evoked by Histamine (100 µM) or ATP (100 µM) (Sigma) as indicated. 30 s before agonist addition drugs were added and maintained during stimulation. In some experiments drugs were added at different time points as specified. At the end of the experiment cells were lysed with 100 µM digitonin in a hypotonic Ca^2+^-rich solution (10 mM CaCl_2_ in H_2_O), thus discharging the remaining aequorin pool. The light signal was collected and calibrated into [Ca^2+^] values by an algorithm based on the Ca^2+^ response curve of aequorin at physiological conditions of pH, [Mg^2+^] and ionic strength, as previously described^56^. In the experiments with permeabilized cells, a buffer mimicking the cytosolic ionic composition (IB) was employed: 130 mM KCl, 10 mM NaCl, 2 mM K_2_HPO_4_, 5 mM succinic acid, 5 mM malic acid, 1 mM MgCl_2_, 20 mM HEPES, and 1 mM pyruvate (pH 7) at 37C. IB was supplemented with either 100 mM EGTA (IB/EGTA) or a 2 mM EGTA-buffered [Ca^2+^] of the indicated concentration (IB/Ca^2+^). HeLa cells were permeabilized by a 1 min perfusion with 100 mM digitonin (added to IB/EGTA) during luminescence measurements. Drugs or DMSO (0.1% v/v) were added to the different perfusion buffers during the entire duration of the experiment. Mitochondrial Ca^2+^ uptake speed was calculated as the first derivative by using the slope Excel function and smoothed for three time points. The higher value reached during Ca^2+^ addition represents the maximal Ca^2+^ uptake speed.

### Metabolomic analysis

ADCM-P and ADCM-D underwent untargeted metabolomics analysis performed by Metabolon, Inc. Briefly, samples were prepared using the automated MicroLab STAR® system from Hamilton Company. Several recovery standards were added prior to the first step in the extraction process for QC purposes. To remove protein, dissociate small molecules bound to protein or trapped in the precipitated protein matrix, and to recover chemically diverse metabolites, proteins were precipitated with methanol under vigorous shaking for 2 min (Glen Mills GenoGrinder 2000) followed by centrifugation. The resulting extract was divided into five fractions: two for analysis by two separate reverse phase (RP)/UPLC-MS/MS methods with positive ion mode electrospray ionization (ESI), one for analysis by RP/UPLC-MS/MS with negative ion mode ESI, one for analysis by HILIC/UPLC-MS/MS with negative ion mode ESI, and one sample was reserved for backup. Based on the metabolomic analysis, a series of metabolites were chosen among the ones that significantly differed from one experimental group to another to be tested in cell cultures, in order to investigate the specific effect of each molecule on mitochondrial calcium transients in both esophageal and breast cancer cell lines.

### Migration assay

MDA-MB-231 cells and OE33 cells were seeded at low confluency (30%) in 6-well plates. 24 h later they were treated with ADCM-P or ADCM-D diluted 1:1 in serum-free medium with or without creatine (100 µM) or creatinine (100 µM) respectively. At the same time a linear scratch was obtained on cell monolayers through a vertically held P200 tip. Images were taken at the indicated time points. ‘‘TScratch’’ software (https://cse-lab.ethz.ch/software/) was used for automated image analysis.

### Clonogenic assay

To evaluate the clonogenic potential, OE33 and MDA-MB-231 cells were seeded (10^3^ cells for each well). 24 h later, ADCM-P or ADCM-D diluted 1:1 in cell medium with or without creatine (100 µM) or creatinine (100 µM) were added respectively.7 days later colonies containing ≥ 30 cells were counted.

### Quantification and statistical analysis

Statistics are reported in the figure legends. All results are representative of at least 3 independent experiments unless otherwise specified and are presented as mean ± SD. Significance was calculated by Student’s two-tailed non-paired t test, ANOVA (one-way or two-way) or by Mann–Whitney Rank Sum Test. All statistical tests were run with SigmaPLot. *P* values < 0.05 were considered statistically significant and marked with asterisks (*p < 0.05; **p < 0.01; ***p < 0.001).

### Study approval

All methods were performed in accordance with the relevant guidelines and regulations. The study related to human adipose-derived conditioned media was approved by Comitato Etico per la Sperimentazione Clinica della Provincia di Padova (Codice CESC 4502/AO/2018) and informed written consent was obtained from all participants.

### Supplementary Information

Supplementary Information 1.

Supplementary Information 2.

Supplementary Information 3.

## Data Availability

The datasets generated and/or analyzed during the current study are available from the corresponding authors on reasonable request**.**
